# Mucormycosis in Patients with Inflammatory Bowel Disease: Case Series and Review of the Literature

**DOI:** 10.1155/2014/637492

**Published:** 2014-04-27

**Authors:** Maheen Z. Abidi, Nayantara Coelho-Prabhu, James Hargreaves, Tim Weiland, Irminne Van Dyken, Aaron Tande, Pritish K. Tosh, Randall C. Walker, Nathan W. Cummins

**Affiliations:** ^1^Division of Infectious Diseases, Department of Medicine, Medical College of Wisconsin, Milwaukee, WI 53226, USA; ^2^Division of Gastroenterology and Hepatology, Mayo Clinic, Rochester, MN 55905, USA; ^3^Altru Medical Center, Grand Forks, ND 58201, USA; ^4^Division of Infectious Diseases, Department of Medicine, Mayo Clinic, 200 First Street SW, Rochester, MN 55902, USA

## Abstract

Mucormycosis is a rare and often fatal invasive fungal infection mostly seen in immune-compromised individuals. A high index of clinical suspicion is necessary, so that effective preemptive therapy can be started, as timely intervention is crucial. In this series we present three cases of invasive mucormycosis in patients with underlying inflammatory bowel disease that had received therapy with immunomodulators prior to the infection. All three had varied clinical manifestations. We also review the literature of invasive mucormycosis in patients with inflammatory bowel disease.

## 1. Background

Invasive mucormycosis (IM) is a rare invasive filamentous fungal infection that is seen primarily in immune-compromised patients [1]. Despite aggressive surgical and antifungal therapy, morbidity and mortality continue to remain high, emphasizing the importance of early clinical suspicion and hence early diagnosis and intervention [2, 3].

Predisposing risk factors that have been described for mucormycosis include chronic high dose corticosteroid use, severe graft versus host disease and its treatment, hematological malignancies, high-risk stem cell transplant, and solid organ transplant recipients [3–7]. Patients with uncontrolled diabetes mellitus and iron overload are also at increased risk for this infection [7].

Patients with moderate to severe inflammatory bowel disease (i.e., Crohn's disease and ulcerative colitis) are typically treated with immunosuppressive agents, including corticosteroids and antimetabolites such as azathioprine. In addition, since tumor necrosis factor-*α* (TNF-*α*) plays a central role in the pathogenesis of Crohn's disease, [8] use of biologic agents, such as monoclonal antibodies inhibiting TNF-*α*, has proven to be effective for this disorder. Use of corticosteroids and TNF-*α* inhibitors places patients at increased risk for opportunistic infections usually seen when cellular immunity is affected, for example, invasive fungal infections [9, 10] and intracellular viruses. The most commonly reported invasive fungal infections in patients with IBD on immunosuppressive regimens have included endemic mycoses, such as histoplasmosis and blastomycosis, and pneumocystis pneumonia. Other invasive mold infections, such as aspergillosis, have also been reported in patients with IBD.

We therefore conducted this case series to study contributing factors, treatment modalities, and outcomes in patients with this rare clinical situation. We report three cases of mucormycosis in patients with IBD collected over a 16-year period (from 1997 to 2013) at Mayo Clinic, Rochester, MN, and review the published literature. This series highlights the severity of this rare infectious complication of IBD.

## 2. Case Reports

Patient 1 was a 43-year-old male who was diagnosed with Crohn's disease of the terminal ileum 9 months prior to presentation. He was initially treated with prednisone starting at 40 mg/day with a 5 mg per week taper. His course was complicated by a perirectal abscess, which was treated with drainage and 1 month of metronidazole. Due to increased gastrointestinal symptomatology, he was restarted on prednisone 25–40 mg daily and azathioprine was added to his regimen 6 months prior to presentation. TNF-*α* inhibitor therapy (adalimumab) was initiated one month prior to presentation. He had no previous history of opportunistic infections.

The patient then presented to an outside hospital with acute fevers, abdominal pain, and hematochezia. WBC was normal, but serum albumin was low (2.7 gm/dL). CT scan of the abdomen demonstrated a significant amount of perirectal air or gas extending along the pelvic sidewall bilaterally and dissecting into the left gluteal musculature. He underwent exploratory laparotomy with small bowel and proximal colon resection with a diverting loop descending colostomy creation. Pathology of the terminal ileum, ileocecal valve, and proximal right colon was consistent with Crohn's disease and perforation of the terminal ileum. He was empirically started on vancomycin, ciprofloxacin, meropenem, and micafungin.

**Figure 1:**
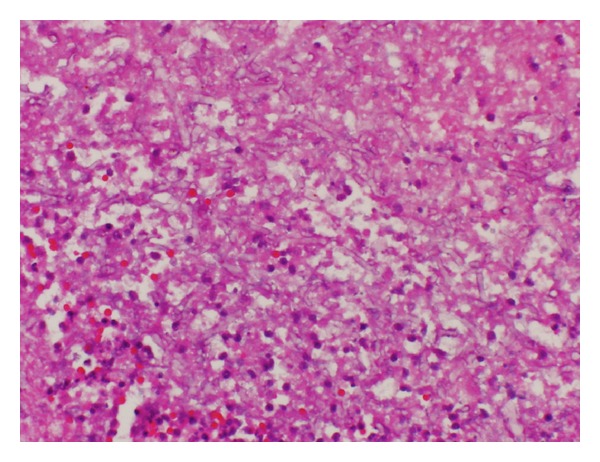
Operatively obtained jejunal tissue section from Patient 3, showing multiple broad hyphae (hematoxylin and eosin, original magnification 400x).

**Figure 2:**
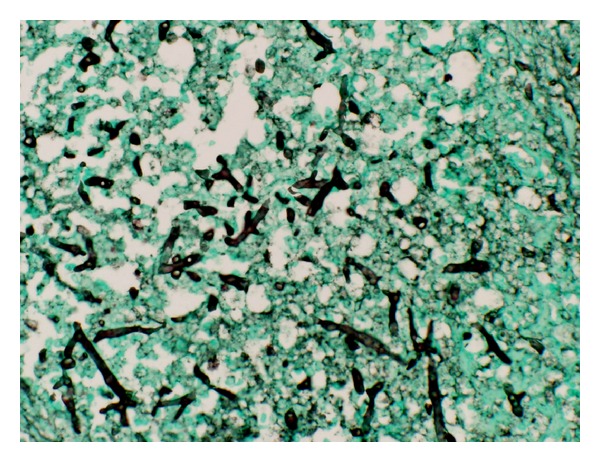
Operatively obtained jejunal tissue section from Patient 3 with fungal elements stained dark against green tissue background (Grocott-Gomori methenamine-silver stain, original magnification 400x).

Blood cultures revealed* Clostridium septicum*. Aerobic and anaerobic abdominal cultures were negative. Repeat exploratory laparotomy demonstrated 7 cm of ischemic proximal jejunum, which was resected. The H&E and silver stain of the jejunum showed 90-degree branching hyphae, slightly wavy, and variable diameter with no definite septa. Focal vascular invasion of fungal hyphae was seen (Figures 1 and 2). Mucoralesdetection by PCR with nested ITS primer at the University of Washington Medical Center demonstrated* Lichtheimia* species (formerly* Absidia* species). Liposomal amphotericin B was added and the patient was transferred to Mayo Clinic.

The patient's immunosuppressants were stopped. Repeat abdominal exploration revealed no evidence of mucormycosis at the jejunal anastomosis, but a large segment of the rectum appeared necrotic and a near-total proctectomy was performed. Pathology did not reveal any evidence of mucormycosis. In the days following surgery, the patient was found to have bleeding near the jejunal anastomotic site treated with clipping and coagulation; there was no endoscopic evidence of residual mucormycosis. Liposomal amphotericin B (7.5 mg/kg) and caspofungin were continued; antibacterial therapy was stopped three weeks from the date of the initial surgery. The patient was started on oral posaconazole once he was reliably able to take oral medications (four weeks after the final GI surgery). Liposomal amphotericin B was stopped two weeks later, once a therapeutic posaconazole level was attained. A 3–6-month course of posaconazole was provisionally considered, provided the patient did not require any further immunosuppression.

The patient had an extended hospital course (101 days) with several complications. He had fistulae between the prostate, urethra, and bladder and a presacral fluid collection draining through the anus requiring bilateral diverting nephrostomy tubes. Additionally, he was found to have multiple abscesses in the abdominal wall and muscles of the legs bilaterally. Cultures from all intraoperative specimens grew* Clostridium septicum* and he was treated with multiple debridements and 5 weeks of piperacillin-tazobactam.

Patient 2 was a 68-year-old woman with a history of chronic ulcerative colitis refractory to medical therapy who underwent total colectomy three years prior to presentation. She also had a history of paroxysmal nocturnal hemoglobinuria requiring long-term corticosteroid therapy, aplastic anemia, and diabetes mellitus. She had no previous history of opportunistic infections. The patient presented initially with a left proximal radial fracture, a left intertrochanteric femoral fracture, and hypothermia having fallen on ice with prolonged environmental exposure. Prior to undergoing operative repair of her fractures, she developed disseminated intravascular coagulation, sepsis, and multiorgan system failure (including pulmonary, renal, and hepatic). Despite appropriate antibiotic coverage for positive blood cultures for* Enterobacter cloacae* and tracheal secretion cultures positive for* Staphylococcus aureus* and* Klebsiella pneumoniae*, the patient developed persistent fever and clinical deterioration. Two weeks after initial presentation, the patient developed a 5 by 6 cm ecchymotic wound of the dorsum of the right hand that progressed rapidly to epidermolysis and necrosis extending to the extensor tendons; it was conjectured at the time that the site was a previous peripheral IV access site, but no clinical documentation confirmed that possibility. Cultures of debrided tissue grew many* Rhizopus* spp. Patient's WBC was elevated at 28,000 WBC/microlitre (4,500–10,000 white blood cells per microlitre around the time she presented with the lesion. Despite surgical debridement and treatment with intravenous amphotericin B deoxycholate the wound infection progressed, with aerial hyphae visible on exam 4 days after initial clinical manifestation. The patient died of progressive multiorgan failure one week later. An autopsy was refused.

Patient 3 was a 30-year-old man diagnosed with Crohn's disease aged 12, who underwent subtotal colectomy with ileostomy and retention of the rectal stump. The patient later developed fistulizing disease complicated by recurrent intra-abdominal abscesses and hospitalizations for sepsis requiring partial small bowel resection. He also suffered from multiple urologic complications, including the development of bilateral nephrolithiasis, ureteral obstruction, and hydronephrosis requiring nephrostomy tube drainage and ureteral stent placement, and both rectovesical and vesicocutaneous fistulae. During a hospitalization for recurrent sepsis, urine cultures obtained from indwelling nephrostomy tubes grew* Acinetobacter*,* Rhodotorula, *and 2 colonies of* Lichtheimia* spp. (an agent of mucormycosis). The patient was not on any immunosuppressive medication at the time, and he had last received TNF-*α* inhibitor therapy (infliximab) nearly one year prior to presentation. He had no history of previous opportunistic infections. The patient was treated with a combination of posaconazole and caspofungin for six months for presumed urinary tract mucormycosis. Repeat urine cultures obtained 3 months into treatment again revealed* Absidia *sp. After six months he underwent operative debridement of a chronic pelvic cavity, cystectomy with ileal conduit urinary diversion, abdominoperineal resection, small-bowel resection, and reconstruction of the pelvic floor. No evidence of active infection was identified at reconstruction, and multiple fungal cultures were negative. He received one additional month of posaconazole postoperatively. The patient suffered from a postoperative bacterial presacral abscess, but no further cultures identified recurrent mucormycosis infection, indicating microbiologic cure of the infection.

## 3. Discussion

Mucormycosis is a rare filamentous fungal infection that is mostly seen in patients in the context of a compromised immune system. While it is an infection occasionally seen in patients with severe neutropenia, it can also be seen in patients with other immune deficiencies. Major risk factors include uncontrolled diabetes mellitus, severe neutropenia, burns and trauma, treatment with corticosteroids, organ and bone marrow transplantation, malignant hematological disorders, and deferoxamine therapy in patients receiving hemodialysis [4]. Clinical manifestations most commonly include pulmonary, isolated sinus, and invasive rhinocerebral and disseminated infections [11]. Urinary, cutaneous, and gastrointestinal manifestations, as presented here, are rare [12, 13]. Attributable morbidity and mortality are high especially with gastrointestinal presentations. GI involvement is acutely and rapidly fatal and hence it is often diagnosed postmortem. Most cases with GI mucormycosis present with symptoms such as GI bleeding, abdominal pain, distension, nausea, and vomiting. The most commonly involved site is the stomach, followed by the colon and the ileum.

We report 3 unusual presentations of mucormycosis in patients with underlying inflammatory bowel disease (IBD) that presented to our institution over time. The first case presented as a case of proven gastrointestinal mucormycosis in a patient with Crohn's disease. Immunosuppressive regimen included prednisone, azathioprine and adalimumab. Most likely this regimen was the etiological factor that led to increased susceptibility to infection by Lichtheimia species. Clinically, the patient improved with surgical debridement and initial therapy with intravenous liposomal amphotericin B and caspofungin. This patient was successfully transitioned to posaconazole on which he remains and continues to do well on follow-up. The second case represents a proven cutaneous mucormycosis in a critically ill patient with a history of ulcerative colitis. Contributing factors to the development of mucormycosis in this patient included corticosteroid therapy for PNH, diabetes, prolonged critical illness, and broad-spectrum antibiotic therapy. Whether a history of ulcerative colitis also played a role is unclear. While the exact cause of death in this patient was unknown due to a lack of autopsy, it is certainly possible that the patient had disseminated mucormycosis given the persistent fever and multiorgan failure despite appropriate broad-spectrum antibacterial therapy. The third case represents a possible case of urinary mucormycosis in a patient with severe fistulizing Crohn's disease.* Lichtheimia *spp. was cultured in separate urinary specimens over a three-month period making culture contamination unlikely. However, definitive disease was not demonstrated by either imaging or pathologic examination. The patient experienced microbiologic clearance with prolonged combination antifungal therapy, and on definitive surgical intervention for his complex urinary disease, no evidence of infection was found. This patient had remote exposure to anti-TNF-*α* therapy; however, his mucormycosis infection was likely a consequence of multiple vesicular fistulae.

A review of the literature reveals 7 previously reported cases of mucormycosis in patients with IBD (Table 1). These were patients with either ulcerative colitis or Crohn's disease, and nearly all were receiving immunomodulator therapy that placed them at increased risk for developing invasive fungal infections. The manifestations were variable, with gastrointestinal presentation being the most common form in three of these cases, all of which subsequently succumbed to the infection and died [12, 14, 15]. Wall and Leman reported a case of sinus mucormycosis that was limited in spread and was noted to respond well to surgical debridement and appropriate antifungal therapy [10]. Follow-up showed the patient to be doing well months after completion of treatment course with antifungals and with cessation of immune suppression. Hunter and Bryant reported a case of cutaneous mucormycosis, which presented as a nonhealing periosteal lesion in a patient with ulcerative colitis who had undergone total colectomy 20 years ago [16]. Cure was achieved by treatment with liposomal amphotericin B and discontinuation of immune suppression. Limited involvement could have played a potential role in recovery from mucormycosis in these two cases. Interestingly, successful treatment was achieved in the only case of mucormycosis endocarditis in this particular group of patients with IBD [17]. Cardiac mucormycosis is extremely rare and is usually reported at autopsy [18]. Early diagnosis and timely intervention with surgical excision of the atrial vegetation, appropriate dosing of liposomal amphotericin B, and discontinuation of corticosteroids aided in recovery of this critically ill patient with cardiac mucormycosis with suspected pulmonary involvement [17]. The most recent case was reported in 2012, which presented as a nonresolving pneumonia in a patient with ulcerative colitis [19]. On bronchoscopy a soft tissue mass was discovered obstructing the bronchus suggestive of either malignancy or invasive mold infection. Despite pneumonectomy and combination therapy with amphotericin B and caspofungin, there was failure to respond to therapy with extensive spread to the contralateral lung and pericardial involvement. Care was withdrawn and the patient died.

Signs and symptoms of mucormycosis can be subtle and nonspecific. Lack of distinctive biomarkers further delays the diagnosis. The beta-D-glucan and galactomannan tests do not detect antigen components from the cell wall of the Mucorales family [3]. A high level of suspicion is needed, keeping in mind that dual infections with other fungal pathogens may be present, as illustrated by our first case [20]. Analysis of specimens from clinically relevant sites is mandatory for diagnosis. Tissue biopsies should be sent for histopathology and culture. Early intervention and multimodal management with repeated surgical debridement and appropriate high dose antifungal therapy with lipid formulations of amphotericin B are the cornerstones of therapy [3]. Optimal daily dose or duration of treatment has yet to be defined. Spellberg et al. describe starting doses of 5–7.5 mg/kg/day for liposomal amphotericin B and 5 mg/kg/day of amphotericin B lipid complex, which is also recommended by 3rd European Conference on Infections in Leukemia (ECIL 3) guidelines on treatment of mucormycosis [7, 21]. For infections involving central nervous system, doses of 10 mg/kg/day for liposomal amphotericin B are suggested [7, 21, 22]. Currently, salvage treatment with 200 mg of posaconazole taken orally four times per day is recommended if the disease is refractory or if there is development of intolerance towards previous antifungal therapy or because of combination of both [21]. No clinical study has evaluated combination posaconazole-polyene therapy for mucormycosis [22]. The pharmacokinetic-pharmacodynamic data for posaconazole raises concerns for achieving adequate blood levels of oral posaconazole for treatment of mucormycosis, making it less likely to be used for combination therapy [22]. ECIL3 recommends the use of combination lipid polyene-echinocandin for salvage therapy after failure of appropriate first line therapy [21]. This is based on synergy seen in murine models of mucormycosis where lipid polyene-echinocandin combination was used [23, 24]. A single retrospective study done in chiefly diabetic patients with rhino-orbital and rhino-orbital-cerebral mucormycosis also demonstrated better treatment success and survival time compared with amphotericin B monotherapy [25]. Surgical debridement remains paramount, and often may need to be both repeated and extensive. Surgery in conjunction with systemic antifungal therapy has been shown to have significantly improved survival [26]. Long-term follow-up is essential to ensure there is no recurrence.

In conclusion, mucormycosis in the setting of inflammatory bowel disease is a rare phenomenon enabled by the use of immunomodulatory agents for IBD, rendering the hosts susceptible to Mucoralesspp. Presentations can be either gastrointestinal of non-gastrointestinal. Mortality rate remains high. Thus, high clinical suspicion and early intervention is needed to reduce mortality associated with this devastating fungal infection.

## Figures and Tables

**Table 1:** Summary of cases of invasive mucormycosis in patients with inflammatory bowel disease described in the literature.

	Case 1	Case 2	Case 3	Case 4	Case 5	Case 6	Case 7
Age (y)/sex	68/M	52/F	41/F	37/M	32/M	59/F	60/M

Risk factor	Ulcerative colitis; diabetes mellitus	Crohn's disease	Crohn's disease; diabetes mellitus (well controlled)	Ulcerative colitis	Crohn's disease	Ulcerative colitis status posttotal colectomy 20 y prior to admission.	Inflammatory bowel disease

Treatment for IBD	Chronic corticosteroids		Azathioprine and infliximab	Corticosteroids, mesalazine	Infliximab infusions, oral 6 mercaptopurine (6 MP), and oral and intravenous corticosteroids	Methotrexate, corticosteroids	Intravenous corticosteroids, oral 6-MP, and oral sulfasalazine

Initial symptoms	Fever, cough, and chest pain. Nonresolving pneumonia	Peritonitis followed by intestinal perforation	Nasal congestion, fullness, headache, and fevers	Exacerbation of underlying condition. In-hospital course: fever, septic shock, trans esophageal echocardiogram: 6 × 1.8 cm right atrial mass	Severe abdominal pain. CT scan abdomen and pelvis: recurrent pneumoperitoneum	Nonhealing periosteal lesion resembling pyoderma gangrenosum	High fever, nausea, vomiting, bloody diarrhea, and pain in right flank

Presentation form of mucormycosis	Pulmonary Bronchoscopy: soft tissue mass obstructing the bronchus intermedius suggestive of fungal pneumonia	Gastrointestinal	Sinus	Endocarditis; suspected hematogenous spread to lungs with multiple nodal lesions with central necrosis on chest computed tomography (CT)	Gastric perforation	Cutaneous	Disseminated (colon and right kidney)

Diagnosis of mucormycosis	Antemortem biopsy of bronchial mass: ulcerated bronchial wall with ischemic necrosis, fibrinopurulent exudates, and hyphae suggestive of mucormycosis	Postmortem: colon tissue: hyphae suggestive of Mucorales, cultures positive for *Rhizopus microsporus* Peritoneal fluid: microscopic exam: broad irregular hyphae, culture: *Rhizopus microsporus *	Antemortem: sinus biopsy: hyphae suggestive of Mucorales	Antemortem: histopathology of atrial endocardial vegetation showed mycotic hyphae. Microbiologic cultures of atrial appendage positive for *Mucor* spp.	Antemortem: abdominal wall: histopathology: many broad based nonseptate hyphae admixed with necrotic tissue.	Antemortem: cutaneous biopsy of new periosteal ulcer: hyphae suggestive of *Rhizopus *spp. on calcofluor white test. Culture of periosteal ulcer grew *Rhizopus* spp.	Antemortem: right kidney: histopathology: large areas of necrosis. Blood vessel walls invaded by hyphae suggestive of Mucorales. Culture of right kidney positive for *Absidia corymbifera *

Treatment	Pneumonectomy Amphotericin B, caspofungin	Voriconazole	Surgical debridement and extensive facial tissue resection. Amphotericin B for 30 days, 6 m course of posaconazole.	Excision of intracardiac mass. Liposomal amphotericin B 5 mg/kg.	Surgical debridement, cholecystectomy. Liposomal amphotericin B (abelcet) 350 mg IV daily	Surgery not done due to medical complications. Liposomal amphotericin B (abelcet). Followed by oral itraconazole for 3 months.	Elective right nephrectomy, total colectomy. Amphotericin B (0.7 mg/kg/d)

Outcome	Worsening of infection while on antifungals with spread to left upper lobe lung and pericardium. Died	Died	Alive, off antifungals, and immune suppression	Alive, off antifungals,and immune suppression	Died	Alive, off antifungals, and immune suppression	Died

Year/reference	2012 [19]	2010 [14]	2009 [10]	2007 [17]	2007 [12]	2002 [16]	1997 [15]
